# Cholesterol-Lowering Effects of Plant Sterols in One Serve of Wholegrain Wheat Breakfast Cereal Biscuits—A Randomised Crossover Clinical Trial

**DOI:** 10.3390/foods7030039

**Published:** 2018-03-16

**Authors:** Peter Clifton, Jennifer Keogh

**Affiliations:** School of Pharmacy and Medical Sciences, Alliance for Research in Exercise, Nutrition and Activity, University of South Australia, Adelaide, SA 5000, Australia; jennifer.keogh@unisa.edu.au

**Keywords:** plant sterols, wholegrain wheat, breakfast cereal, crossover clinical trial, LDL cholesterol

## Abstract

The meta-analysis of plant sterol supplement studies suggests an 8% lowering of low density lipoprotein (LDL) cholesterol for 2 to 2.5 g/day of plant sterols. Cereal foods have been rarely tested, and one study showed a lower LDL lowering of 5.4% with 1.6 g of plant sterol in breakfast cereal. We aimed to test a breakfast wheat biscuit with 2 g of plant sterols in a single serve of two wholegrain wheat breakfast cereal biscuits. Fifty volunteers with a total cholesterol of >5.5 mmol/L were recruited for a randomised crossover study with two 4-week periods with no washout, of which 45 successfully completed the study. After exclusion of four outliers, the difference in LDL cholesterol between standard wholegrain wheat breakfast cereal biscuit and plant sterol-enriched wholegrain wheat breakfast cereal biscuit was 0.23 mmol/L or 5.6% (*p* = 0.001) with a 95% confidence interval of 2.4–8.9%. Men and daily cereal consumers had greater responses 9.8% vs. 3.6% and 7.2% vs. 3.8% respectively (*p* < 0.05). The LDL lowering effect of 2 g of plant sterol enriched from one serve of wholegrain wheat breakfast cereal biscuit was not significantly different from other food products delivering 2–2.5 g of plant sterols daily. Regular cereal consumers have a better response.

## 1. Introduction

Plant sterols and stanols have been tested in over 124 trials with 201 test arms [1]. This meta-analysis showed an 8.0% (95% CI: 9.0, 7.0) lowering of LDL cholesterol in 40 plant sterol studies with a dose of 2–2.5 g (average sterol dose of 2.1 g/d). Solid forms of sterol-containing foods showed a 9% (95% CI: 10.8, 7.2) lowering of LDL cholesterol. A previous cereal study with 40 people consuming 45 g of cereal containing 1.6 g of sterol (as esters) showed an average lowering of LDL cholesterol of 5.4% (*p* < 0.05 versus a control cereal) each consumed for 3 weeks in people with an average LDL cholesterol of 4 mmol/L [2]. This result was lower than expected, although similar to that seen with bread in the same study (6.5% lowering of LDL). One other study has examined rye bread containing 2 g of plant sterols and demonstrated an 8% lowering of LDL cholesterol [3]. We aimed in this study to investigate the cholesterol-lowering effects of 2 g of plant sterols in one serving of a wholegrain wheat biscuit to expand the evidence concerning the effectiveness of sterol-enriched cereals. The single serve formulation is a novel way of achieving the recommended intake of 2 g of plant sterols/day [4].

## 2. Methods

*Volunteers.* We aimed to complete the study with 40 volunteers, as this would give us 80% power to see a 5% difference in LDL cholesterol (*p* < 0.05). We recruited 50 volunteers on the assumption that 10% would drop out.

Inclusion criteria: age 18–75 years, body mass index (BMI) < 30 kg/m^2^, total serum cholesterol >5.5 mmol/L and <7.5 mmol/L, no lipid lowering medication, no diabetes, normal thyroid status and no metabolic disorder other than hyperlipidaemia, not taking medications likely to affect lipid metabolism and no requirement for such medication, serum triglycerides <4.5 mmol/L, no strong aversion and no known allergies/intolerances to wheat.

Volunteers were screened with point of care finger-prick testing for cholesterol in the fasting state using an Accutrend plus device (Roche, Basel, Switzerland).

**Figure 1:**
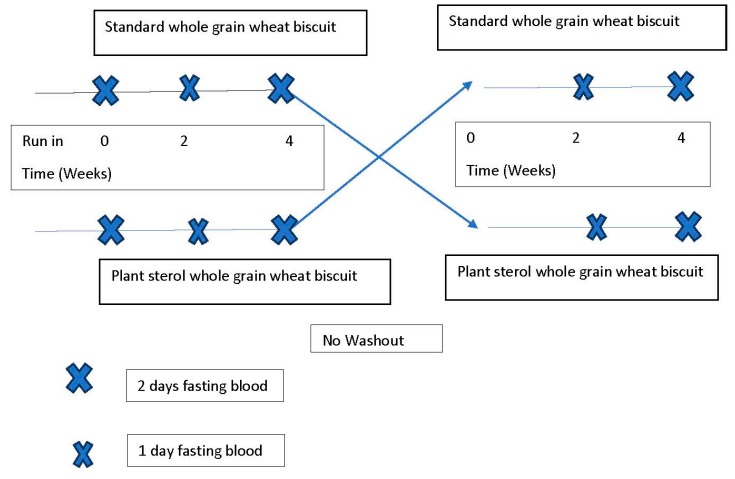
Study design.

Trial design. The trial was a double-blind randomised crossover study with no washout (Figure 1). A run-in phase of 2 weeks on usual foods was followed by 2 randomised 4-week intervention phases with standard wholegrain wheat breakfast cereal biscuit (supplied as Weet-Bix by Sanitarium Health and Wellbeing, Berkeley Vale, NSW, Australia, composition 97% wholegrain, 0.8% calories as fat, 3.5 g fibre and 91 mg sodium per serve) or plant sterol-enriched wholegrain wheat breakfast cereal biscuit which contained 2 g of plant sterols (as esters) per serve, with one serve being 2 biscuits (each biscuit delivered 1 g of plant sterols). During the 4-week intervention phases, participants were instructed to eat one serve per day. During the run-in phase, a 3-day weighed food record was taken to establish a baseline diet, and fasting lipids and glucose on 2 days were taken at the end of this time. At the end of each 4-week phase, volunteers had fasting lipids taken on 2 days with a single fasting test for lipids at 2 weeks. Three-day weighed food records (2 weekdays and 1 weekend day) were taken during each phase, as well as daily records of wholegrain wheat breakfast cereal biscuit consumption and a count of remaining wholegrain wheat breakfast cereal biscuit. Weight was measured at each visit. Plant sterol-enriched and standard wholegrain wheat breakfast cereal biscuit were packed in plain, opaque packing coded A and B and were indistinguishable. The company held the code until the analysis had been done and report had been written. Wholegrain wheat breakfast cereal biscuits were planned to be consumed in a standard way with 120 mL 2% fat milk if the volunteers were agreeable. If volunteers chose alternative ways of consuming wholegrain wheat breakfast cereal biscuit this was repeated in both phases. Volunteers were asked to keep their diets the same across both phases. Volunteers were asked to bring back the wholegrain wheat breakfast cereal biscuit box from the first phase before being given the next box of cereal for the second phase and asked to keep a checklist of cereal consumption in each phase. Volunteers were asked about usual patterns of cereal consumption and the form in which the test cereal was consumed.

Written informed consent was obtained from all participating volunteers. The study was conducted in accordance with the Declaration of Helsinki, and the protocol was approved by the Ethics Committee of the University of South Australia (Project identification code 0000035456) on 9 June 2016.

### 2.1. Blood Samples

Blood for serum was collected in a tube with no additives and the tube was kept upright in a tube rack at room temperature for 30 min to ensure complete clot formation and then placed on ice. Blood for plasma was collected in a sodium fluoride EDTA tube, and the tube was placed immediately on ice until centrifugation and processing. Blood samples were centrifuged at 4000 RPM at 4 °C for 10 min (Universal 32R, Hettich Zentrifugen, Tuttlingen, Germany). Plasma glucose, fasting serum total cholesterol (TC), triglyceride (TG), high-density lipoprotein cholesterol (HDL-C, Direct method) were measured using an automated spectrophotometric analyzer (Konelab 20XTi, Thermo Electron, Waltham, MA, USA). Low density lipoprotein cholesterol (LDL) was calculated (LDL = TC − TG × 0.45 − HDL) [5].

### 2.2. Statistics

All data shown is mean plus standard deviation. Data quality was assessed by examining the distribution of the change in LDL cholesterol between baseline and regular wheat biscuit and between baseline and sterol-enriched wheat biscuit. Data distribution was assessed using Kolmogorov–Smirnov and Shapiro–Wilk tests of normality and probability plot (PP) and quantile quantile (QQ) plots against a normal distribution to highlight any outliers. Outliers were also assessed using box plots with those >quartile 3 + 1.5 × the interquartile range or <quartile 1–1.5 × the interquartile range. The quartiles were based on Tukey’s hinge values. Once any outliers were removed and the data was assessed to be normal in distribution the primary analysis was a repeated measures ANOVA adjusting for baseline lipids and diet order. Significance was accepted as *p* < 0.05.

## 3. Results

Fifty people were enrolled in the trial, 25 in treatment order 1 and 25 in treatment order 2. Three people failed to commence the trial and one dropped out during the first phase. Forty-six people successfully completed the trial protocol. One was excluded for carryover of supplies from one phase to the other, so 45 people were evaluable. This group included 19 men and 26 women aged 57.8 ± 14.3, BMI 24.9 ± 3.5, screening total cholesterol 6.42 ± 0.63 mmol/L and baseline LDL cholesterol of 4.23 ± 0.87 mmol/L.

Compliance was excellent. Twenty-one out of 45 consumed only 1–2 more (or less) than expected, while 24 consumed exactly the number expected over the entire trial period. Overall, the average number consumed per phase was exactly as expected for both standard and plant sterol-enriched wholegrain wheat breakfast cereal biscuit. Twenty-one consumed it with milk alone, six with yogurt with or without fruit and two consumed it with spread or dry. All the rest consumed it with milk plus fruit or yogurt or other cereals and seeds. Twenty-five consumed it whole and 20 crushed. Full cream milk was used by 10 and the other 28 consumed a variety of milks. Participants could not identify the cereal containing sterol (47% in error). Weight did not change significantly over the 10 weeks from 69 kg at baseline to 68.9 kg at the end of the study with no differences from the beginning to the end of either diet period. There were no dietary changes or weight changes across the three treatment periods. Fibre, fat, protein, carbohydrate and energy did not change with the introduction of wholegrain wheat biscuit in the two intervention phases, however wholegrain intake increased by 33% (*p* < 0.01) and there were increases in thiamin (37%, *p* < 0.001), riboflavin (28%, *p* < 0.001), niacin (13%, *p* < 0.01) and iron (27%, *p* < 0.001) from the run in phase diet.

### 3.1. Lipids

Four outliers were removed because of large rises in LDL cholesterol from the baseline value, two on standard wholegrain wheat breakfast cereal biscuit and two on plant sterol-enriched wholegrain wheat breakfast cereal biscuit.

### 3.2. Total Cholesterol

Total cholesterol fell by 0.28 mmol/L or 4.4% (Table 1) with a treatment effect *p* = 0.002, treatment by diet order *p* = 0.085 (*n* = 41, 22 order 1 and 19 order 2). There was an effect of gender *p* = 0.004 which improved the treatment significance (*p* < 0.0001). In males (*n* = 12) TC was lowered by 0.64 ± 0.65 mmol/L and in females (*n* = 21) by 0.14 ± 0.40 mmol/L (9.5% vs. 2.1% *p* = 0.004).

### 3.3. LDL Cholesterol

The treatment effect was highly significant with a *p* = 0.002 and the diet order by treatment was not significant (*p* = 0.2). LDL cholesterol fell by 0.01 ± 0.37 mmol/L compared with baseline on standard wholegrain wheat breakfast cereal biscuit when consumed first and by 0.13 ± 0.28 mmol/L when consumed second suggestive of a non-significant carryover effect of the plant sterol into the second phase. The difference from baseline of the LDL following sterol-enriched wholegrain wheat breakfast cereal biscuit was the same whether consumed first or second at 0.33 ± 0.41 and 0.27 ± 0.46 mmol/L. Thus, the placebo adjusted change is 0.23 ± 0.44 mmol/L or 5.6%. The 95% confidence interval of the mean change was 0.10–0.37 mmol/L (2.4–8.9%). Those who had a rise in LDL cholesterol from baseline on plant sterols (the opposite to the expected fall or no change =12) also had a rise in LDL on placebo (*n* = 8 of the 12) with a net rise on plant sterol treatment of 0.08 mmol/L (not significant), so clearly there is a large amount of noise in the cholesterol measurement. Consumers who regularly consumed cereal on a daily basis prior to the trial had a greater response than those who were not, −7.2% vs. −3.8%, and men were predominant in this group, and this alone explained the gender effect. On backward regression, regularly consuming cereal (*p* = 0.006), age (inversely *p* = 0.010), triglycerides at baseline (inversely *p* = 0.025), % carbohydrate at baseline (inversely 0.04) and whole or crushed (whole better 0.017) were significant with an adjusted r squared of 0.28 (*p* < 0.001). Neither gender nor baseline LDL remain in the equation.

### 3.4. Triglycerides and HDL

Neither HDL cholesterol or triglyceride changed significantly.

## 4. Discussion

We have shown that one serve of wholegrain flaked wheat biscuit enriched with 2 g of plant sterols lowers LDL cholesterol by 5.6% (*p* < 0.002) with 66% of participants showing an LDL cholesterol lowering. This result was the same as our previous cereal study, which had a similar baseline LDL cholesterol (about 4 mmol/L) but used a totally different cereal formulation, which achieved an LDL cholesterol-lowering of 0.24 mmol/L (95% CI 0.13–0.35) and a confidence interval similar to the current trial. Given the pooled statin studies results, we would expect that an average lowering of LDL cholesterol by 0.23 mmol/L would reduce major cardiovascular events over 5 years by about 5% [6], but of course this proposition cannot be tested without a very large trial. For instance, the “Improve it” study required over 18,000 patients with an acute coronary event followed for over 7 years to show an absolute 2% difference in primary endpoints for a 0.4 mmol/L difference in LDL cholesterol [7]. The Cholesterol Treatment Triallists Collaboration [6] is a meta-analysis at the individual patient level of 27 statin trials in 134,537 individuals, which found that a 1 mmol/L reduction in LDL cholesterol reduced the risk of vascular disease by 21% largely regardless of age, gender, baseline LDL cholesterol or history of vascular disease. The “Improve it” study [7] added 10 mg ezetimibe or placebo to 40 mg of simvastatin in patients with an acute coronary event and demonstrate a 15% reduction in the risk of the primary cardiovascular endpoint.

If we compare our results to the values from the Ras meta-analysis [1] for the dose range of 2.0–2.5 g (average 2.1 g) of sterols studies only, then the 5.6% lowering of LDL we saw was not significantly different from the 8% (95% CI: 9.0, 7.0) lowering seen in the 40 sterol studies in this meta-analysis (*p* = 0.2) which however were mostly studies using spreads and dairy foods, which required participants to consume the product multiple times over the day. The only other cereal study in the literature using a high fibre rye bread demonstrated an 8.1% lowering of LDL cholesterol after 4 weeks [3]. A cereal bar study using 2 g of plant sterols showed a lowering of LDL cholesterol of 5.4% after 3 weeks [8].

Although we saw age and gender effects in this study, these did not occur in the meta-analyses [1,9], so it is difficult to know their meaning in our population, although gender could be eliminated when other factors where entered into the backward regression. However, we did not see an effect of baseline LDL cholesterol, which is unusual given that we had a similar range of cholesterol values in the participants as in other trials. A larger population may illuminate some of these issues. The non-responders in this population would be accounted for by two populations—one in which cholesterol absorption was inhibited but cholesterol synthesis increased and LDL cholesterol did not change. The second population is those who had a rise in LDL on both sterol wheat biscuit as well as regular wheat biscuit. This is most likely just experimental noise rather than an over-exuberant reactive rise in cholesterol synthesis in the liver increasing LDL cholesterol, as the regular wheat biscuit also had a rise in LDL cholesterol. In many dietary studies, a non-response rate of 30–50% is seen mainly because random experimental noise overwhelms any small effect of diet, and because it is random it will vary with time and is not predictable.

The observation about responders regularly consuming cereal is novel. This suggests that the microbiome may be playing a previously undetected role in cholesterol absorption or rapidly scavenging cholesterol displaced from the micelle and enhancing the effectiveness of plant sterols. In addition, the inverse relationship with percentage dietary carbohydrate at baseline suggests that fibre-rich cereal carbohydrate may be a major contributor to the diet in responders rather than free sugars or low fibre carbohydrate. Propionate may be the key molecule produced by the microbiome that reduces lipogenesis and serum cholesterol and wheat fibre-derived arabinoxylan maybe a key substrate [10]. Feeding rats a combination of mixed lactobacilli and 5% cellulose lowered LDL cholesterol [11] while feeding plant sterol ester led to major shifts in the microbial population [12], possibly by providing additional cholesterol for bacterial growth. In a stanol-feeding study, Baumgartner and others [13] found a relationship between several microbial species and serum phytosterol levels, suggesting there is an interaction between sterol absorption (both cholesterol and phytosterol) and the microbiome.

## 5. Conclusions

The LDL lowering effect of 2 g of plant sterol from one serve of wholegrain wheat breakfast cereal biscuit was not significantly different from other food products delivering 2–2.5 g of plant sterols daily. Two g of plant sterol in one serve of wholegrain wheat biscuits lowered LDL cholesterol by 5.6% after 4 weeks, but, given the confidence interval, the true population effect could be as high as 9%. Thus, using wholegrain wheat breakfast cereal biscuit is a convenient, easy and nutritious way to achieve the 2 g/day of plant sterol intake, and its form lends itself to excellent daily compliance.

## Figures and Tables

**Table 1:** Lipid levels.

Lipid Species	Baseline	Week 2 Regular Biscuit	Week 4 Regular Biscuit	Week 2 Plant Sterol Biscuit	Week 4 Plant Sterol Biscuit	Plant Sterol Biscuit Difference from Baseline	Difference between Plant Sterol and Regular Biscuit
TC	6.26 ± 0.78	6.30 ± 0.79	6.31 ± 0.85	6.13 ± 0.84	6.03 ± 0.87	0.24 ± 0.54 * 3.8 ± 8.8% *	0.28 ± 0.53 * 4.4 ± 8.1% *
LDL	4.21 ± 0.84	4.17 ± 0.88	4.14 ± 0.88	4.00 ± 0.81	3.91 ± 0.88	0.30 ± 0.43 * 7.1 ± 10.6% *	0.23 ± 0.44 * 5.6 ± 1.0% *
HDL	1.61 ± 0.51	1.65 ± 0.49	1.70 ± 0.54	1.71 ± 0.53	1.69 ± 0.54	−0.07 ± 0.18	0.01 ± 0.19
TG	0.98 ± 0.39	1.13 ± 0.45	1.05 ± 0.47	0.97 ± 0.40	0.96 ± 0.37	0.02 ± 0.23	0.09 ± 0.23

Lipid results in 41 subjects (mmol/L) after exclusion of 4 outliers. * *p* < 0.05. Data was analysed with repeated measures ANOVA adjusted for baseline values and treatment order. TC: total cholesterol, LDL: low density lipoprotein cholesterol, HDL: high density lipoprotein cholesterol, TG: triglyceride.
